# No relationship between researcher impact and replication effect: an analysis of five studies with 100 replications

**DOI:** 10.7717/peerj.8014

**Published:** 2020-03-24

**Authors:** John Protzko, Jonathan W. Schooler

**Affiliations:** Psychological and Brain Sciences, University of California, Santa Barbara, Santa Barbara, CA, United States of America

**Keywords:** Reproducibility, Bibliometrics, Metascience, *h*-index, Registered Replication Reports, Scientometrics, Hypothesis Testing, Laboratories, Replication Crisis, Expertise

## Abstract

What explanation is there when teams of researchers are unable to successfully replicate already established ‘canonical’ findings? One suggestion that has been put forward, but left largely untested, is that those researchers who fail to replicate prior studies are of low ‘expertise and diligence’ and lack the skill necessary to successfully replicate the conditions of the original experiment. Here we examine the replication success of 100 scientists of differing ‘expertise and diligence’ who attempted to replicate five different studies. Using a bibliometric tool (*h*-index) as our indicator of researcher ‘expertise and diligence’, we examine whether this was predictive of replication success. Although there was substantial variability in replication success and in the h-factor of the investigators, we find no relationship between these variables. The present results provide no evidence for the hypothesis that systematic replications fail because of low ‘expertise and diligence’ among replicators.

## Introduction

Scientific findings cannot exist in isolation, but rather must rely on the capacity of other laboratories to successfully replicate them. When only the ‘discovering’ lab can replicate a result, trust in the finding can and should be examined. As a consequence of increased concern regarding the replicability of scientific results, showing the same results given the same methods with new data, psychologists have initiated assorted replication efforts to assess the reliability of extant research findings. The results of these large-scale replication attempts have introduced new questions into the field. One such initiative ran single replications of 100 studies and reported only about one third of the studies replicated according to various plausible criteria for what should count as a successful replication (Open Science Collaboration, 2015; see also Earp, 2016). While conclusions regarding the actual replication rate in this and other efforts have been debated (e.g., Gilbert et al., 2016a; Gilbert et al., 2016b; Anderson et al., 2016; Etz & Vandekerckhove, 2016), the question of *why* systematic replication efforts have routinely failed to replicate original findings has become an important topic in psychology.

There are a number of reasons why efforts to replicate a research finding may fail. A relatively rare reason is that researchers deliberately fabricate the initial result (see John, Loewenstein & Prelec, 2012; Fiedler & Schwarz, 2016; and Agnoli et al., 2017 for admission rates). A more commonplace source of false findings is engaging in selective reporting of participants, conditions, or analyses (e.g., Simmons, Nelson & Simonsohn, 2011; see also John, Loewenstein & Prelec, 2012) that can make non-results appear real. If an original effect is wholly fallacious, it is easy to explain why it would not replicate. But what about findings that corresponds to real effects?

There are also a number of reasons why replication efforts may fail to replicate a real effect including lack of power in the replications (Cohen, 1969), lack of fidelity among researchers to the procedures of the original study (see Gilbert et al., 2016b), unacknowledged variance in auxiliary assumptions (Earp & Trafimow, 2015), deliberate questionable research practices used by the replicator to show a lack of evidence (e.g., Protzko, 2018), among others.

In this manuscript, we investigate a separate possible explanation, researcher ‘expertise and diligence’. Related to the notion of replication fidelity, it seems reasonable that highly skilled researchers would be more effective than less skilled researchers in isolating the dependent and independent variables in a manner that enables the original finding to be replicated. An apocryphal example comes from cognitive dissonance research, where different researchers read the same exact script to participants to induce a feeling of choosing to do the researcher a favor, yet different abilities in acting and expressing sincerity would represent a lack of fidelity if replicating experimenters do not come off as sincere to participants as the original authors. If processes such as these alter replicability, then such a capacity to effectively carry out the replicated research might be expected to be associated with the achievement of the investigator carrying out the replications. In other words, researchers who have been highly successful in carrying out their own lines of research may be better able to effectively carry out replications than their less successful counterparts.

The above logic suggests researchers who engage and fail in replicating canonical studies are of inferior ‘expertise and diligence’ (Bartlett, 2014; Cunningham & Baumeister, 2016). In this study, we test this hypothesis using the *h*-index of replicators in a series of pre-registered replications to determine whether researchers of higher ‘expertise and diligence’ are more successful at replicating a given effect. Notably, this hypothesis regarding the relationship between replication and *h*-index has been put explicitly forth:

“In other words, it is much easier to be a successful nonreplicator while it takes ‘expertise and diligence’ to generate a new result in a reliable fashion. If that is the case, it should be reflected in measures of academic achievement, e.g., in the *h*-index or in the number of previous publications.” (Strack, 2017; p. 9).

Although various researchers have speculated on the role of researcher ‘expertise and diligence’ in replication success, the only (indirect) empirical evidence to speak to this question comes from a re-analysis of 100 single replications of prominent psychology findings (Open Science Collaboration, 2015). Specifically, the number of times a study was internally replicated by the original authors in the original publication was not predictive of whether an outside research team could replicate the effect (Kunert, 2016, cf. Cunningham & Baumeister, 2016). While this was interpreted as evidence for the prevalence of questionable research practices (a researcher who engages in such practices to ‘create’ an effect is likely to do it in many of their own internal replications), the evidence could also support the hypothesis that the original authors had the requisite ‘expertise and diligence’ the replicating team lacked. Both interpretations rely on a property of the original research team (tendency to engage in QRPs, special ability) that was not shared by the replicating team.

As the hypothesis has been put forward that researchers of different degrees of ‘expertise and diligence’ (indexed through *h*-index) are more or less able to experimentally replicate an effect, we sought to address the question empirically. Given previous speculations that replication failures are a product of a lack of adequate skill set on the part of researchers, and that the *h*-index is a reasonable metric by which to assess researchers’ acumen (especially as put forward in the hypothesis to be tested), we sought to test this conjecture. As replication ‘success’ is a function of the observed effect size and the sample size, given that the studies we investigate here have a more fixed sample size, we investigate the hypothesis in the context of the observed effect size returned by a replicator as a function of their *h*-index (a proxy for ‘expertise and diligence’ outlined above).

### Replications

To test the hypothesis that replication success is a function of researcher ‘expertise and diligence’, we collected 100 replications that had been conducted across five studies. We used the first five published Registered Replication Reports (RRRs), investigations where a dozen or more individual research teams all attempt to replicate the same study.

RRRs are direct replications of a study conducted by multiple, independent laboratories of researchers who vary in the extent to which they believe in the original finding. All labs follow the exact same protocol approved by the original study team or by surrogates who share their theoretical perspective before data collection begins.

The five RRRs represent multi-lab replications of the following phenomena: verbal overshadowing (Schooler & Engstler-Schooler, 1990); priming commitment and reaction to hypothetical romantic betrayal (Finkel et al., 2002); the facial feedback hypothesis (Strack, Martin & Stepper, 1988); ego depletion (Hagger et al., 2016), and that people are intuitively cooperative yet deliberatively selfish (Rand, Greene & Nowak, 2012). All data and analysis scripts are archived at https://osf.io/qbq6v/.

The reason for using this as our sample was that it provided multiple replications of the same basic effect by researchers of varying levels of ‘expertise and diligence’. In one replication investigation (Open Science Collaboration, 2015), researchers who had more publications chose to replicate studies with larger original effect sizes (Bench et al., 2017). After taking this initial volunteering into effect, there was no residual relationship between researcher ‘expertise and diligence’ and replication success (cf. Cunningham & Baumeister, 2016). As that investigation only looked at one replication per study, however, it was unable to look at replication variation within the same study. The analysis proposed here is able to look at variation in ‘expertise and diligence’ across different replicators within multiple replications of the same study. Instead of one effect having one replication and averaging across different studies, each study under replication has multiple effect sizes from multiple researchers. In short, by examining the replication success of multiple investigations of the same effect, it should be in principle possible to isolate the role of variation in researchers’ ‘expertise and diligence’ in contributing to their effectiveness in replicating the original findings.

### Researcher ‘expertise and diligence’

We used the *h*-index of the researchers who undertook replications of the various effects (following the hypothesis of Strack, 2017 and the previous work of Cunningham & Baumeister, 2016). The *h*-index for a given scientist is a function of the number of papers that author has published and the number of times those papers have been cited. Thus, it is more sensitive to research impact than simply measuring the raw number of publications. It is important to point out that the *h*-index is a transformation of the number of times that a research has been cited (Hirsch, 2005). The correlation between a researchers *h*-index and the total number of times they have been cited, for example, is *r* = .96 (Ruscio et al., 2012). Thus, the results here would be nearly identical if we used raw number of citations (or, for example, raw number of publications). Typical h-indices for psychological scientists range from six to ten (Ruscio et al., 2012).

Previous research into the replicability success of different research teams have used number of publications as a measure of “high-expertise” (Cunningham & Baumeister, 2016, p. 12; citing Bench et al., 2017). The *h*-index also incorporates a function of the number of times that research has been cited. Thus, it incorporates impact within a field and the number of impactful papers a researcher has published (see Nieminen et al., 2006 for an insightful critique). It is a better metric of researcher ‘expertise and diligence’ than number of publications, which is more aptly characterized as researcher productivity (Hirsch, 2005). A researcher who publishes many papers that are largely ignored by their scientific peers would have a low *h*-index.

Although *h*-index is arguably the best standardized measure of researcher ‘expertise and diligence’ available, it is possible that it fails to capture the critical dimensions of researcher ‘expertise and diligence’ that could in principle underpin replication success. Nevertheless, given that *h*-index has been previously hypothesized to be an appropriate metric of researcher ‘expertise and diligence’ that could account for variations in replication, it is important to rigorously test it. Continually shifting the definition of researcher ‘expertise and diligence’ to other metrics may start to introduce operationalism into the hypothesis and make convergence on a solution impossible. Thus, we directly test the conjectures of prior authors and examine the extent to which researchers with different h-indices vary in their replication success.

## Materials and Methods

### Verbal overshadowing RRR

The original finding under investigation was verbally describing a previously seen face causes a decrease in participants’ ability to accurately identify the face in a subsequent lineup (Schooler & Engstler-Schooler, 1990). 23 separate labs engaged in a replication of this study (Alogna et al., 2014; study 2). The 23 labs were able to successfully replicate the result in the resulting meta-analysis.

Heterogeneity in meta-analyses can indicate the possibility of result moderation (see Higgins & Thompson, 2002). The 95%CI of heterogeneity was 0% to 46%. Implicit in this and all subsequent analyses is the presumption that there is some threshold of heterogeneity adequate for testing the researcher ‘expertise and diligence’ hypothesis. Any threshold would, by definition, be arbitrary. If the RRRs truly have zero heterogeneity, failures to replicate cannot be explained away by differences in researcher ‘expertise and diligence’ As this would also require accepting the null of ‘no heterogeneity’, we forego any such accepting and pursue the analyses as they are.

### Priming commitment RRR

The original finding under investigation was inducing commitment in relationships by either describing how partners are ‘linked’ versus describing how partners are independent causes people to be more forgiving of hypothetical betrayals (Finkel et al., 2002, study 1). 16 separate labs engaged in a replication of this study (Cheung et al., 2016). There were four dependent variables used in the replications. We chose the one most consistent with the original authors’ hypothesis and that showed the largest amount of potential heterogeneity (neglect responses) for investigation here. This variable is also most central to the claim of the original publication. These neglect responses are passive ways of trying to undermine a relationship, such as giving someone the ‘cold shoulder.’ Thus, the original finding and one under investigation here is that people would show less neglect responses to a hypothetical betrayal if they had been primed with commitment. The 16 labs were unable to successfully replicate the commitment priming on neglect responses, either as individual labs or when meta-analytically combined. The 95% CI of heterogeneity was 0% to 54%. Therefore, there exists the possibility that differences in researcher ‘expertise and diligence’ can explain the variation in effect sizes and the failures to replicate.

### Facial Feedback RRR

The original finding under investigation was that making people smile by holding a pencil pointing upwards in one’s mouth versus downwards caused them to find humorous cartoons more humorous (Strack, Martin & Stepper, 1988, study 1). 17 separate labs engaged in a replication of this study (Wagenmakers et al., 2016). The 17 labs were unable to successfully replicate the result, either individually (no statistically significant effects) or when meta-analytically combined (again no statistically significant effect). The 95% confidence interval for degree of heterogeneity was 0% to 51%. Therefore, there exists the possibility that differences in researcher ‘expertise and diligence’ could explain the variation in effect sizes.

### Ego Depletion RRR

The original finding under investigation was that engaging in a difficult cognitive control task (versus not engaging in a difficult task) depletes people’s resources and hampers performance on a subsequent task (increased reaction time variability; Sripada, Kessler & Jonides, 2014). The difficult task was watching a series of words on a video screen and pressing a button when a word with the letter “e” in it was displayed, withholding the response if the “e” was next to or one letter away from a vowel. The subsequent task was a response inhibition task. 23 separate labs engaged in a replication of this study (Hagger et al., 2016). The 23 labs were unable to successfully replicate the result either individually or when meta-analytically combined. The 95% confidence interval for degree of heterogeneity was 0% to 45%. Therefore, there exists the possibility that differences in researcher ‘expertise and diligence’ can explain the variation in effect sizes.

### Intuitive Cooperation RRR

The original study under investigation involved playing a Public Goods Game where participants were able to cooperate with others by donating more or less of their endowed money to a public pot to then be shared among all the players. The original study found that forcing people to decide quickly (<10 s) how much of their endowment to contribute gave more money than those who were forced to wait (Rand, Greene & Nowak, 2012). 21 separate labs engaged in a replication of this study (Bouwmeester et al., 2017). The 21 labs were unable to successfully replicate the result either individually or when meta-analytically combined using an intention-to-treat (ITT) analysis. For the ITT analysis the 95% confidence interval for degree of heterogeneity was 0% to 47%. Therefore, there exists the possibility that differences in researcher ‘expertise and diligence’ can explain the variation in effect sizes.

### Overall analyses

To test the researcher ‘expertise and diligence’ and replicability hypothesis, we collected the raw effect sizes from each of the five above-mentioned RRR. We then matched the obtained effect size with the senior author of the replication’s *h*-index. We chose to use the highest *h*-index within a team of researchers as our metric to prevent the possibility that all of our data would be aggregated at the level of students within the lab.

**Figure 1:**
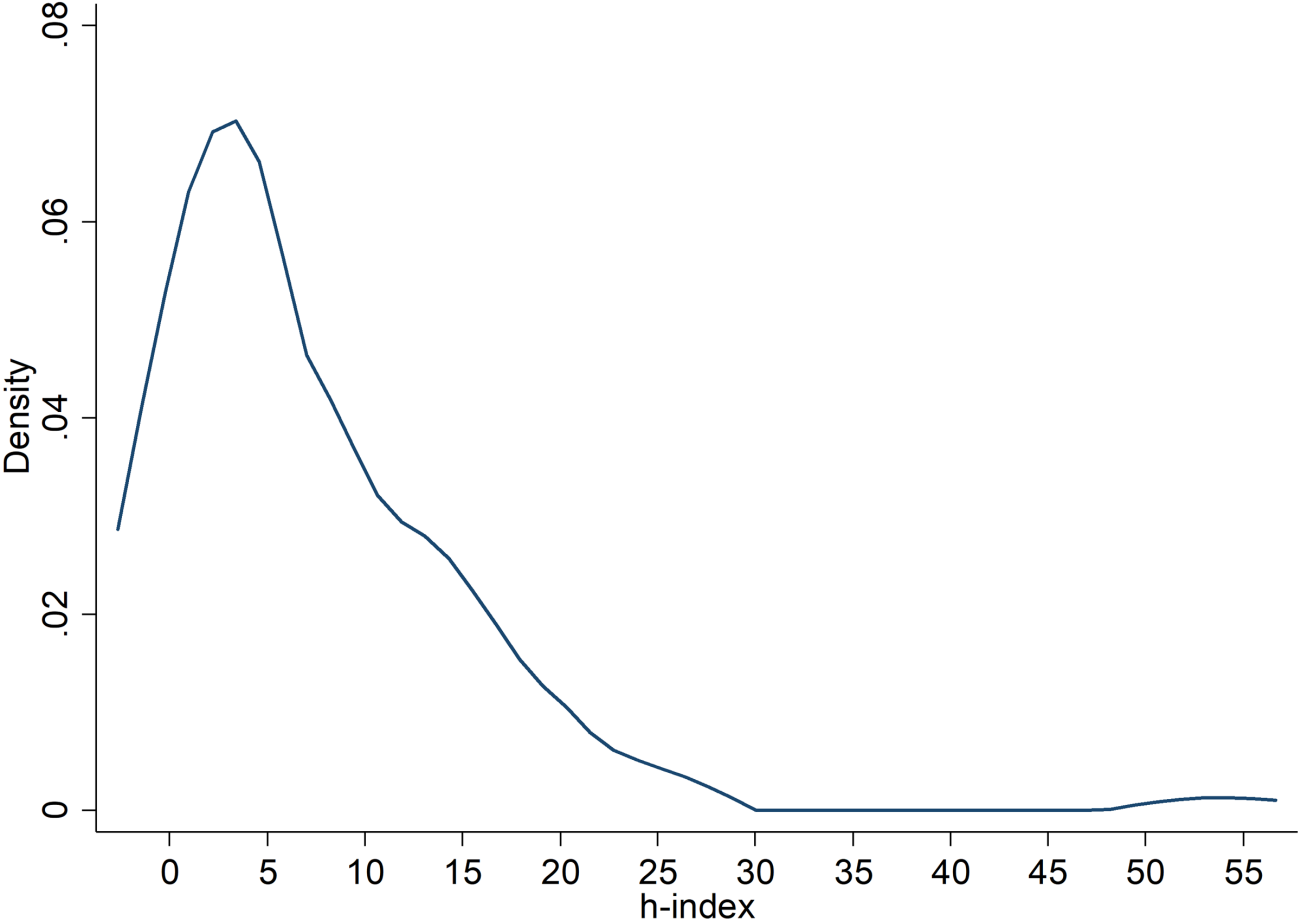
Kernel density plot of the distribution of h-indices of the first authored researchers contributing replication effect sizes to this study.

These *h*-indexes were collected from GoogleScholar and when none was available, using Web of Science between October 3-17, 2016, on the same date as often as possible. To test the hypothesis, we used meta-regression with the obtained effect size, weighted by the meta-analytic standard error for each replication, testing whether researcher ‘expertise and diligence’ was associated with the obtained effect size (Cohen’s **d**). This analysis is similar to a variance-weighted least squared regression that does not assume homogeneity of variance and adds in the estimate of between-study variability (*τ*^2^) into the error variance component to the meta-analysis. Then, researchers’ *h*-index is used as a predictor variable for the dependent variable (replication effect size). There was sufficient variation in *h*-indexes (0 to 54) to allow for testing this prediction; the average *h*-index was 7, with a standard deviation of 8, and a median of 4 (see Fig. 1).

As supplementary and exploratory analyses, we also test the researcher ‘expertise and diligence’ hypothesis using the average *h*-index of all authors listed on each replication. All analyses were run in STATA 13.1 using the metareg command, unless otherwise noted. We first present the analyses for each individual RRR, followed by a pooled analysis.

## Results

### Verbal overshadowing

**Figure 2:**
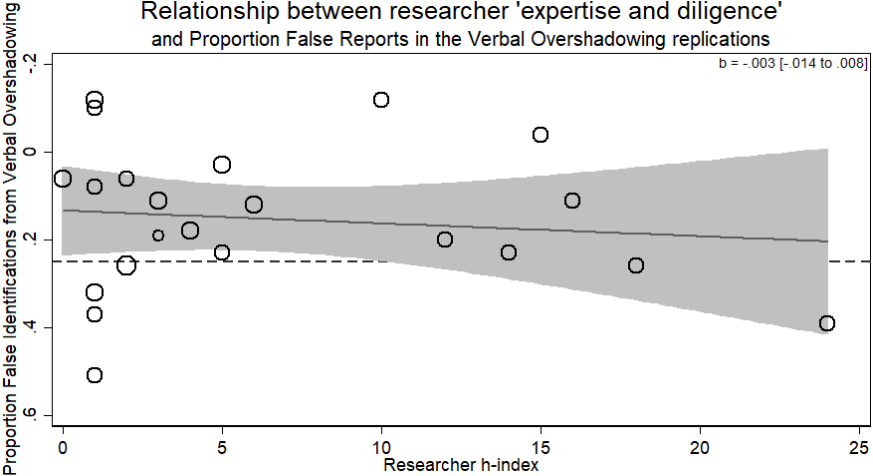
Meta-regression of researcher ‘expertise and diligence’ on obtained effect size in replicating the verbal overshadowing paradigm. Point estimates weighted by the inverse of the standard error, so larger circles indicate replications with greater weight. Dashed horizontal line represents magnitude of original effect of Verbal Overshadowing. Note *y*-axis is in reverse scale to represent the hypothesized direction of results.

There was no evidence for the researcher ‘expertise and diligence’ and replicability hypothesis. Specifically, more experienced researchers (those with a higher *h*-index) got the same results as less experienced researchers (*b* =  − 003, *p* > .59, 95% CI [−.014–.008]). Thus, while verbal overshadowing was successfully replicated, variation in the magnitude of the effect was not related to researcher ‘expertise and diligence’ (see Fig. 2).

The supplementary, exploratory analysis using the average *h*-index of all listed authors and not simply the first author from each lab showed a significant *negative* relationship between average *h*-index and effect size (*b* =  − .013, *p* = .035, 95% CI [−.024 to −.001]). One should be careful about interpreting this negative association between researcher ‘expertise and diligence’ and replication success. While this observation certainly does not support the hypothesis of a positive association, the statistical evidence is weak and quite possibly a type I error (or a Type M error of exaggerated estimates in low power situations; Gelman & Carlin, 2014).

### Priming commitment

**Figure 3:**
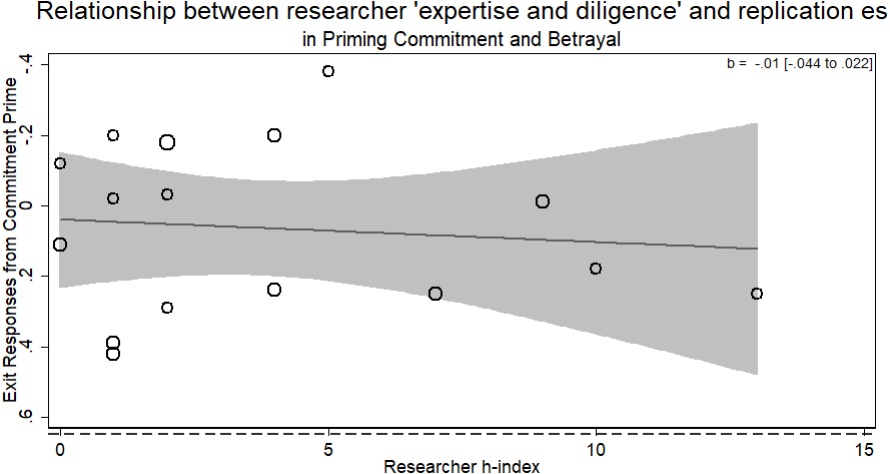
Meta-regression of researcher ‘expertise and diligence’ on obtained effect size in replicating the priming commitment and betrayal paradigm. Point estimates weighted by the inverse of the standard error, so larger circles indicate replications with greater weight. Dashed horizontal line represents magnitude of original effect of priming Commitment (*d* = .65). Note *y*-axis is in reverse scale to represent the hypothesized direction of results.

There was no evidence for the researcher ‘expertise and diligence’ and replicability hypothesis. Specifically, more experienced researchers were just as likely to return the same effect size as novice researchers (*b* =  − .01, *p* > .5). Across the different laboratories attempting the replication, those who returned larger effect sizes were of no different ‘expertise and diligence’ than those who returned smaller ones (see Fig. 3). There was only one author listed on each replication in this RRR.

### Facial feedback

**Figure 4:**
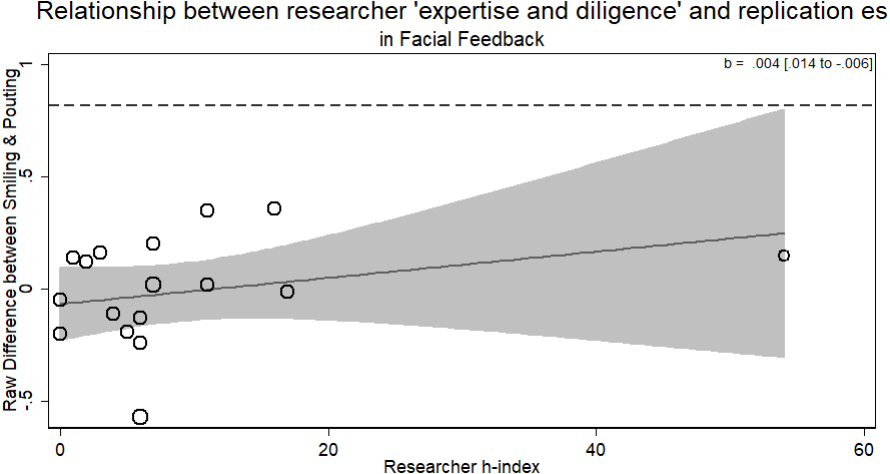
Meta-regression of researcher ‘expertise and diligence’ on obtained effect size in replicating the facial feedback paradigm. The analysis with removing the researcher with an *h*-index of 54 (as it may be an outlier) does not change the results (*b* = .014, *p* > .33). Point estimates weighted by the inverse of the standard error, so larger circles indicate replications with greater weight. Dashed line represents magnitude of original effect.

There was no evidence for the researcher ‘expertise and diligence’ and replicability hypothesis. More experienced researchers (with a higher *h*-index) were just as likely to return the same effect size as novice researchers (*b* = .004, *p* > .33). The overall result of the RRR was unable to replicate the Facial Feedback hypotheses. Within the different laboratories attempting the replication, those who returned an effect consistent with the original hypothesis were of no different level of ‘expertise and diligence’ than those who did not (see Fig. 4). There was only one author listed on each replication in this RRR.

### Ego depletion

**Figure 5:**
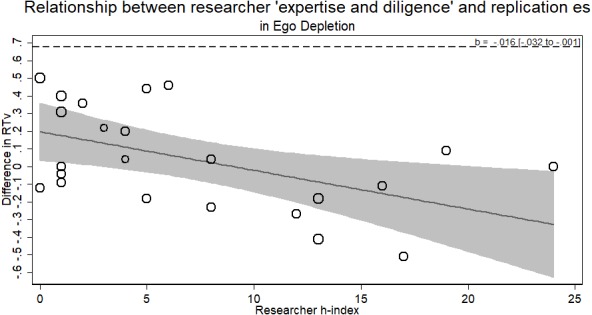
Meta-regression of researcher ‘expertise and diligence’ on obtained effect size in replicating the ego depletion paradigm. Point estimates weighted by the inverse of the standard error so larger bubbles indicate studies with greater weight. Dashed line represents original effect size in reaction time variability (RTv).

The ego depletion RRR sample provides a different result from the previous samples. Unlike before, we did observe a relationship between researcher ‘expertise and diligence’ and the observed effect of exerting a large amount of mental control decreasing performance on a subsequent task. The results, however, ran *counter* to the researcher ‘expertise and diligence’ and replicability hypothesis. Researchers with more ‘expertise and diligence’ actually observed smaller, indistinguishable from zero effects (failing to replicate), whereas more novice researchers observed larger effects of depletion (*b* =  − .016, *p* < .044; see Fig. 5).

Though against the hypothesis, the significance of this relationship raises three vexing alternatives. Either: (a) ego depletion by this paradigm is not a real effect and we have evidence that higher ‘expertise and diligence’ researchers can confirm it; (b) ego depletion by this paradigm is a real finding but it can only be found by researchers of low ‘expertise and diligence’ (cf. Cunningham & Baumeister, 2016); (c) the positive effects observed with the lower ‘expertise and diligence’ researchers were simply a type 1 error. We take no firm stance here and believe future replication efforts of ego depletion using multiple labs of a variety of ‘expertise and diligence’ should be conducted using a different paradigm to elucidate this result. Furthermore, using the average *h*-index of all listed authors in the exploratory analysis showed no relationship between average author *h*-index and replication effect size (*b* =  − .003, *p* > .66).

### Intuitive cooperation

The Intuitive Cooperation RRR presents as a more difficult case than the other four RRRs. Re-analyzing the data by dropping those who did not comply to the time constraints led to a successful replication of the main finding (see also Rand, 2017). We address the non-compliance in a manner different from the original authors. The original authors dropped all participants who did not comply with the time constraints to achieve a complier-only analysis. The problem with this approach is that it ignores possible differences between those who comply and those who do not. Furthermore, as the two conditions of ‘wait’ and ‘choose fast’ represent different designs, the ‘type’ of participant who complies may be different between the groups (e.g., more intelligent participants better able to comply in the fast condition but equally able to comply in the wait condition). At worst, this approach can lead to comparing one type of participants against a different type of participants, breaking randomization and defeating the possibility of drawing strong causal conclusions. An easy analogue to this is drug trials. While it may seem appropriate in a drug trial with a passive control group to drop participants assigned to take the drug but never did, doing so means you are comparing the whole control group against just the more conscientious members of the drug group.

Thus, we present the instrumental variable (i.e., ‘encouragement design’, see Gelman & Hill, 2006) first. Then, we analyze the data as the intention-to-treat analysis, a more conservative approach that retains the causal inference warrant of randomization. Finally, we report the association of researcher ‘expertise and diligence’ on these instrumental variable estimates. As there was only one author listed on each replication in this RRR, we pursue no additional average *h*-index analysis.

**Figure 6:**
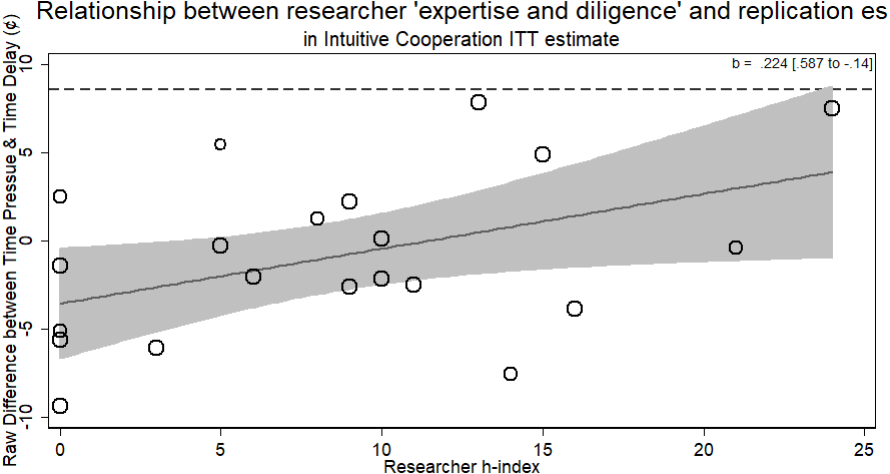
Meta-regression of researcher ‘expertise and diligence’ on obtained effect size in replicating the intuitive cooperation paradigm using the raw observed differences. Point estimates weighted by the inverse of the standard error. Dashed line represents original study effect.

More experienced researchers (with a higher *h*-index) were just as likely to return the same effect size as novice researchers (*b* = .224, *p* > .21). The overall result of the RRR was unable to replicate the Intuitive Cooperation hypothesis. Within the different laboratories attempting the replication, those who returned an effect consistent with the original hypothesis were of no different level of ‘expertise and diligence’ than those who did not (see Fig. 6).

### Instrumental variable analysis

The primary data from each lab was collected from https://osf.io/cmu34/. Individuals at the participant-level were binary coded for whether they complied with the time constraints (<or >10 s based on speeded or delay condition, respectively). This was used as a metric of treatment compliance. A two-stage least squared regression was calculated for each replication in STATA using the *ivreg* command. Detailed results from this analysis are available on the OSF page.

Treating the Intuitive Cooperation as a randomized encouragement design where participants were encouraged to decide quickly or slowly provides the strongest test of the hypothesis, as it does not suffer from the conservativeness of the ITT analysis but preserved randomization. From this analysis, we can see that the effect of deciding *quickly* how much money to contribute to the public pot for those who did so when encouraged versus deciding slowly when encouraged to led people to contribute a non-significant 1.378% more amount of money (95% CI [5.517 to −2.76]).

**Figure 7:**
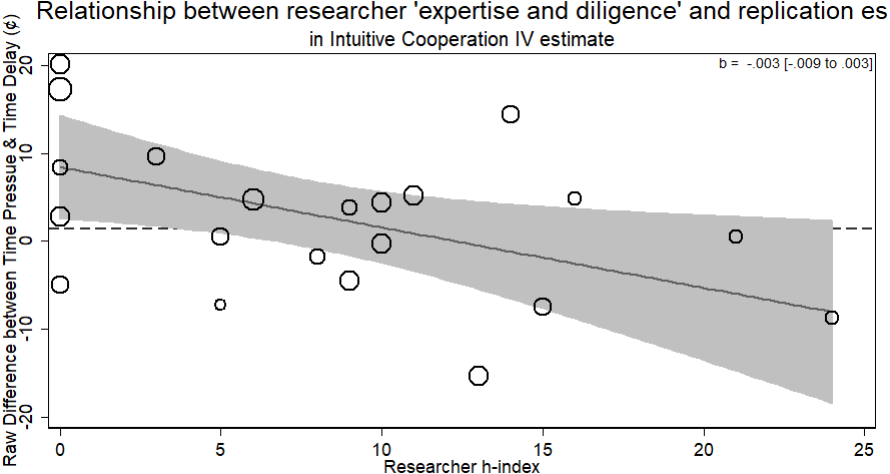
Relationship of researcher *h*-index and instrumental variable estimate of group contributions on estimated compliance. Bubbles are weighted by the inverse ivreg standard error. Dashed line represents original study result.

Using these estimates, we can now see whether researchers of different levels of ‘expertise and diligence’ were able to return different effect sizes. The standard errors from this meta-regression were the standard errors of the estimates from the instrumental variable regression. The results from this analysis showed no evidence for the researcher ‘expertise and diligence’ and replicability hypothesis. Researchers who had more cited publications returned effect sizes that were no different in magnitude than novice researchers (*b* =  − .003, 95% CI [−.009–.003], *p* > .26; see Fig. 7). Thus, researchers with more ‘expertise and diligence’ got the same rates of compliance and the same effect sizes.

Thus, for both the instrumental variable and the ITT results, we see no solid evidence that researchers with a higher *h*-index returned different effect sizes from their replications of the intuitive cooperation RRR.

### Overall results

In each of the RRRs reported so far, there is no evidence for the researcher ‘expertise and diligence’ and replicability hypothesis. It is possible that within each paradigm the effect was underpowered (see Hedges & Pigott, 2004). Therefore, as a robustness check, we combined the first four RRRs and tested once again the hypothesis that returned effect size in a replication is a function of the experience the researcher brings to the experiment.

We omitted including the intuitive cooperation RRR from this analysis as the measure used was substantively different from the other RRRs (% money instead of a quantitate value not bounded by 0 or 1). This would not change the results; however, as the slope for the instrumental variable analyses that take into account compliance is in the direction against the researcher ‘expertise and diligence’ and replicability hypothesis (and nearly perfectly flat, see Fig. 7). Thus, its inclusion would only further push the estimate away from the hypothesized direction. Furthermore, as an exploratory analysis suggested by one reviewer, there was no evidence for a quadratic relationship between researcher ‘expertise and diligence’ and returned effect size either (*p* > .45).

In RRRs where the original result was negative in value, confirmation of the researcher ‘expertise and diligence’ hypothesis would indicate a negative slope to the regression of ‘expertise and diligence’ on effect size, with ‘better’ researchers contributing effect sizes further away negatively from zero (Verbal Overshadowing; Priming Commitment). In RRRs where the original result was positive, however, the predicted slope would be positive, with larger effect sizes from researchers with more ‘expertise and diligence’ (Facial Feedback; Ego Depletion). As this combination of positive and negative slopes could cancel each other out, we switched the sign of the effect sizes from Verbal Overshadowing and Priming Commitment so the overall prediction would indicate a positive slope.

**Figure 8:**
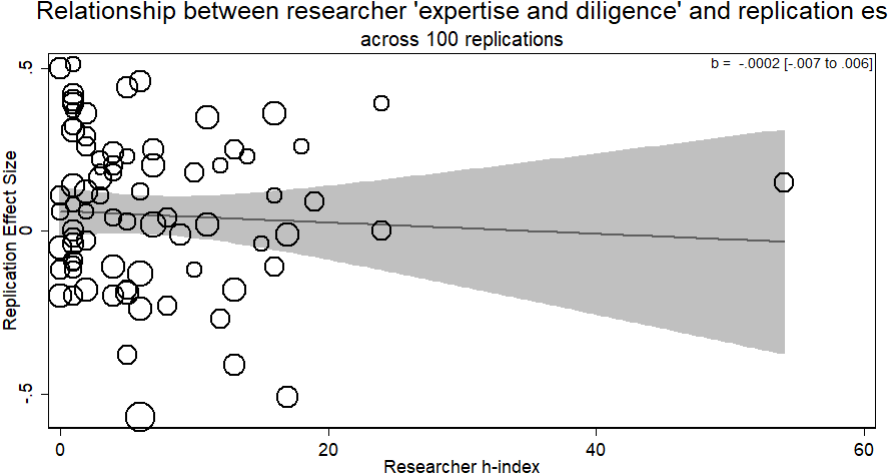
Meta-regression of researcher ‘expertise and diligence’ on obtained effect size across replications in all four RRRs. Studies are re-coded so higher-expertise and diligence researchers should have larger effects in the positive direction. Bubbles are weighted by the inverse of the standard error so larger bubbles exert more weight.

The results further supported the conclusion that there is no empirical support for a relationship between researcher ‘expertise and diligence’ and replicability in the studies investigated here. Simply pooling all data together showed no evidence of a relationship between the ‘expertise and diligence’ of the researchers in these four RRRs and the absolute value of the effect sizes they returned (*b* =  − .0002, *p* > .93; see Fig. 8). These null results were the same when including dummy variables for each of the studies contributing to the overall analysis (e.g., dummy variable for effect sizes coming from the Verbal overshadowing RRR, Facial Feedback RRR, Ego Depletion RRR) to control for differences in the average effect size measured by different RRRs (*b* =  − .002, *p* > .69). This dummy coding was done due to the low number of clusters or studies would prevent accurate estimation of clustering. One possible concern was that there was one researcher with an *h*-index of 54 who represents a statistical outlier from the rest. However, running the same meta-regression with that outlier removed does not change the results (*b* = .001, 95% CI [−.006–.006], *p* > .96); there was still zero relationship between researcher’s *h*-index and their returned effect size in a replication. As two further exploratory analysis suggested by reviewers, the results remain the same when analyzed as an unweighted regression (*b* (75) = −.0006, *p* > .83, 95% CI [.005–−.007]); and assuming the *h*-index is an ordered categorical variable and not a quantitative measure without the assumption of equidistance returns a null result (*b* = .328, *p* > .51).^1^ 1This analysis was done by switching the *h*-index and returned effect size in the model using the omodel command in STATA (Wolfe & Gould, 1998; Long & Freese, 2006) which treats the dependent variable as an ordered categorical variable without the proportional odds assumption.Experimenters who had not a single cited study before were just as likely to converge on the same meta-analytic effect as those who had published numerous, highly-cited studies. Finally, as the combined results are negative in slope, increasing the power of the analysis would not show any effect for the researcher ‘expertise and diligence’ and replicability hypothesis.

Furthermore, a lack of statistical significance is not enough to fully conclude that there is no relationship between two variables. One such approach is to use equivalence testing, testing whether the observed effect is equivalent to a hypothesis of ‘zero effect’ or an exceptionally small effect size (see Lakens, in press). To do so, we calculate the 90% confidence interval around the estimate (90% CI [−.005–.005]) to test the equivalence of these results with a true null effect. This analysis tests whether the estimate is indeed statistically indistinguishable from a ‘smallest effect size of interest’, and provides an interpretation to null effects. Thus, unless the theory is that each increase in a researchers ‘expertise and diligence’, indexed by their *h*-index, is associated with an increase in effect size of .005 or smaller (upper bound of the 90% CI for the equivalence test), we can say the results here are equivalent to a zero effect.

### Mean differences and precision

Effect sizes in these instances are a function of the mean difference between two groups divided by the precision of their estimate. It could be the case that researchers of higher ‘expertise and diligence’ get more precise estimates (smaller standard deviations holding sample size constant)—not necessarily larger group differences. For this to be the case, however, there would need to be a *negative* relationship between ‘expertise and diligence’ and mean differences. Holding mean differences constant, if precision increased (standard deviations decreased) then effect sizes would go up. The only way for the ‘precision’ effect to be possible, considering the above (largely) null effects on effect sizes, would be smaller mean differences mixed with estimates that are more precise across ‘expertise and diligence’. A supplementary meta-regression showed there is no relationship between group mean differences and ‘expertise and diligence’ in any study (all *p*s > .385). Thus, there could not be a ‘precision’ effect—however measured—without showing either an effect size effect or a mean differences effect.

## Discussion

The question of whether ‘expertise’ in psychology predicts replication success (Strack, 2017) is one that is important to understand when considering the implications of large-scale replication efforts. If more novice researchers of lower ‘expertise and diligence’ are unable to find basic canonical effects when replicating, it is extremely important to consider such information.

Here we used a metric of researcher ‘expertise and diligence’ to look at replication effect size among labs conducting the same studies. The hypothesis has been explicitly put forward that researchers fail to replicate a finding because they are of lower ‘expertise and diligence’. As our analysis uses variation within the same replication effort, there is the increased chance to find evidence for the researcher ‘expertise and diligence’ hypothesis as different replicators may or may not return different effect sizes. Our results showed no evidence whatsoever in favor of the researcher ‘expertise and diligence’ and replicability hypothesis. In four of the five RRRs, there was no association between obtained effect size and the ‘expertise and diligence’ of the researcher conducting the replication. In one of the RRRs, we actually saw evidence that more experienced researchers were closer to returning the overall meta-analytic effect of zero, with less experienced researchers being the ones who found evidence for ego depletion. Collapsing across all RRRs, the relationship was zero (*b* = .00003, *p* > .992).

Thus, researcher ‘expertise and diligence’ did not predict replication effect size regardless of whether the studies replicated the effect. The central claim of the researcher ‘expertise and diligence’ and replicability hypothesis is that failed replications by researchers of low ‘expertise and diligence’ have no bearing on the truth of the effect, because only researchers of high ‘expertise and diligence’ are able to show the effect. In the analyses in this paper, however, there was no evidence of a relationship between the hypothesized researcher ‘expertise and diligence’ and their ability to replicate an effect. This was also true in the situation where the researchers were able to replicate the effect (Verbal Overshadowing); even in this case, researcher ‘expertise and diligence’ did not matter.

We agree wholeheartedly with the sentiment that “metascience is not exempt from the rules of science” (Gilbert et al., 2016a, p. 1037-a). As such, we sought to empirically test the belief among some researchers that replicability is a function of the ‘expertise and diligence’ of the one conducting the study, as opposed to letting the hypothesis stand untested. Such a hypothesis deserves empirical attention as replication becomes more common within psychological science.

Since this investigation used multiple labs replicating the same studies, our results are unbiased by publication bias or missing studies. A significant concern in meta-analyses of extant literature is that non-significant effects find are less likely to be published and thus less likely to be included in meta-analyses. In the RRRs, however, the entire pool of replication studies was analyzed and published regardless of outcome. Consequently, the meta-analytic effect size from these studies is unaffected by publication bias.

It is of course challenging to guarantee the fidelity of replication efforts, which is why it seemed plausible that researcher ‘expertise and diligence’ could have affected replication success. In this context, it is notable that, all research teams attempted to follow the exact same protocol and the original authors approved the replication materials (or by surrogates who share their theoretical perspective). Furthermore, there was sufficient variation among indices of researcher ‘expertise and diligence’, as well as sufficient variation of effect sizes to permit accurate testing. In addition, there was enough heterogeneity within each RRR to allow the possibility that differences in returned effect size could be a function of something other than random chance. Researcher ‘expertise and diligence’ was not it.

The RRRs we used ranged from those that successfully replicated the original finding (Alogna et al., 2014), to those that successfully replicated the manipulation but failed to provide evidence for the outcomes (Hagger et al., 2016), to those which were unable to replicate the basic manipulation (Cheung et al., 2016). Therefore, there was a range of possibilities that could have arisen, including different relationships between researcher ‘expertise and diligence’ and effect sizes in different paradigms. That the only relationships to emerge was one backwards to the hypothesis under test (that higher ‘expertise and diligence’ researchers were less likely to replicate the result of the ego depletion study) stands as evidence against the researcher ‘expertise and diligence’ and replicability hypothesis.

## Limitations

The *h*-index is not without its problems as a metric of researcher ‘expertise and diligence’ (e.g., Yong, 2014). However, few if any other objective measures could be considered better. This could plausibly limit the generalization unless a better metric if found. The problem with defining researcher ‘expertise and diligence’ is avoiding circularity. If only researchers of high ‘expertise and diligence’ can replicate ego depletion, for example (see Cunningham & Baumeister, 2016), then how can we define ‘high expertise and diligence’ outside of ‘is able to replicate ego depletion’? We believe the *h*-index provides an objective metric that combines number of publications and impact of those publications. As such, it avoids such circularity (see Barnes, 2016; Barnes, 2017 for further information on the *h*-index debate). Better metrics could be used in the future with further large-scale replication attempts to tease apart the nature of any relationship, although none was found here.

The results here could also call into to question the relevance of the *h*-index as a valid marker of researcher ‘expertise and diligence’. Although the motivation for this example of testing a metascientific claim involves the explicit prediction that researcher *h*-index (Strack, 2017) or its analogue number of citations (Cunningham & Baumeister, 2016); which *h*-index correlates with at .96; (Ruscio et al., 2012) validly marks ‘expertise and diligence’, such scientometrics may not index the ‘right’ kind of skills.

The *h*-index will vary, for example, depending on the area of research of an author, does not control for self-citation, and is blind to the role the co-author had in the actual publications—it is the same for being skilled or merely for providing useful structures and materials. In short, the link between *h*-index and a researcher’s ability to conduct good studies is hypothetical, and it could be at best an imperfect one^2^. 2We thank Dr. Danielle Fanelli for bringing this argument and the one above to our attention.It is this explicit hypothesis, however that we sought to test. It would be desirable to eventually develop alternative metrics of ‘ability to conduct good studies’ to put similar unenumerated hypotheses to the test. Until then, such unenumerated alternate metrics of ‘expertise and diligence’ can be considered speculative at best.

Unrelatedly, it may be the case that there was a low number of researchers of high ‘expertise and diligence’ in these replications. As can be seen in Figs. 2–6, there tended to be a larger number of researchers with lower *h*-indices. Thus, the situation here could be a case of incomplete coverage of a moderator. While this may be true in the individual study analyses, in the aggregated analysis there is sufficient coverage to suggest this is not an issue overall.

Finally, it should be acknowledged that a failure to find a relationship between researcher ‘expertise and diligence’ and replication success does not rule out implementation fidelity as a factor in replications failures. Indeed in at least two of the five replication efforts examined here, fidelity issues in the approved protocols may have contributed to replications outcomes. In the case of RRR1, 31 labs were actively collecting data when it was discovered that the timing parameters in the approved replication protocol significantly deviated from those of the original study the project was trying to replicate. As a result, a second replication version of the study with a corrected protocol (including 21 labs) was carried out. Verbal overshadowing was replicated with the corrected protocol but not with the initial one (see Schooler, 2014, for discussion). Had this deviation in the fidelity of the procedure not been addressed, a faulty conclusion regarding the robustness of verbal overshadowing would have been made. Relatedly, with respect to RRR3, one study (Noah, Schul & Mayo, 2018) provided some evidence that a key difference between the original paradigm and the replication effort (videoing participants) could account for the disparate outcomes. Although the importance of this disparity can be contended (the difference between the original and videoed conditions was only marginally significant), this study highlights the possibility that seemingly modest procedural changes may contribute to replication failures. Thus, while at present there is no evidence that researcher ‘expertise and diligence’ contributes to the success of replications efforts; there is evidence that the fidelity of the approved replication protocol can be critical. Furthermore, if the work of the editorial staff for the psychology RRRs turns the novice replicators into effective experts by buffering their ‘inability’, then there is an immediate tension; the results from the individual RRRs cannot now be invalid because novices undertook them. Either the RRR meta-analytic results hold for the studies conducted thus far because all involved researchers are turned into effective experts via the work of the editorial staff and the effect represents what is true in the world (often null); or the effect of the original study is still trustworthy but researchers of higher ‘expertise and diligence’ cannot find it any better than ones of lower ‘expertise and diligence’.

## Conclusion

The researcher ‘expertise and diligence’ and replicability hypothesis is an attractive one in interpreting large-scale failures to replicate. While it may be tempting to dismiss such a hypothesis outright, metascience is not exempt from empirical testing. The results here showed no evidence for researcher ‘expertise and diligence’ hypothesis to explain the failures to replicate numerous RRRs or even when the RRR is successful (see Verbal Overshadowing). Beyond statistical non-significance, the results from the combined analysis of four of the RRRs explored was equivalent to a True effect of zero. Thus, we have directed an empirical test of the hypothesis to date and found it lacking evidence. It may well be the case that in some situations researcher ‘expertise and diligence’ underpins investigators ability to replicate findings, however, the present findings suggest that such a mechanism was not involved in one of the most ambitious series of replication efforts to date.

## Additional Information and Declarations

### Data Availability

The following information was supplied regarding data availability: The raw data is available at the Open Science Framework: Protzko, and Jonathan Schooler. 2019. “No Relationship between Researcher ‘expertise and diligence’ and Replicability: An Analysis of Five Studies with 100 Replications.” OSF. August 18. https://osf.io/qbq6v/.
